# Acceptability of an integrated school-based HPV vaccination program within two districts of Tanzania: A qualitative descriptive study

**DOI:** 10.1371/journal.pgph.0001394

**Published:** 2023-01-04

**Authors:** Dominique Guillaume, Joseph G. Rosen, Linda B. Mlunde, Belinda J. Njiro, Castory Munishi, Davis Mlay, Amelia Gerste, Taylor A. Holroyd, Mary Rose Giattas, Christopher Morgan, Bruno F. Sunguya, Furaha Kyesi, Florian Tinuga, Joseline Ishengoma, Rupali J. Limaye

**Affiliations:** 1 International Vaccine Access Center, Johns Hopkins University, Baltimore, Maryland, United States of America; 2 Jhpiego, Johns Hopkins University affiliate, Baltimore, Maryland, United States of America; 3 Center for Infectious Disease and Nursing Innovation, School of Nursing, Johns Hopkins University, Baltimore, Maryland, United States of America; 4 Department of International Health, Bloomberg School of Public Health, Johns Hopkins University, Baltimore, Maryland, United States of America; 5 School of Public Health and Social Sciences, Muhimbili University of Health and Allied Sciences, Dar es salaam, Tanzania; 6 School of Population and Global Health, University of Melbourne, Victoria, Australia; 7 Ministry of Health, United Republic of Tanzania; 8 President’s Office Regional Authority and Local Government, United Republic of Tanzania; 9 Department of Epidemiology, Bloomberg School of Public Health, Johns Hopkins University, Baltimore, Maryland, United States of America; 10 Department of Health, Behavior & Society, Bloomberg School of Public Health, Johns Hopkins University, Baltimore, Maryland, United States of America; University of Cape Town, SOUTH AFRICA

## Abstract

Tanzania has one of the highest cervical cancer incidence and mortality rates in sub-Saharan Africa. The Tanzanian Ministry of Health developed an integrated adolescent health program, *HPV-Plus*, that combines HPV vaccination with additional health services: nutritional assessments, vision screening, and vaccination for adolescent girls, and education for all genders. This qualitative descriptive study evaluated the acceptability of the *HPV-Plus* program in two districts in Tanzania. Key informants comprising of adolescent girls, parents, program planners, and program implementers in Njombe and Dar es Salaam Tanzania were interviewed to assess the program acceptability. Transcripts were analyzed using a team-based iterative thematic analysis approach, consisting of both inductive and deductive coding. The Theoretical Framework of Acceptability was used to guide analysis, with themes categorized according to theoretical constructs of intervention coherence, affective attitudes and perceptions, and perceived effectiveness. Overall acceptability of the *HPV-Plus* program was high among stakeholders. The most salient finding regarding factors that influenced HPV vaccine acceptability was largely related to education and knowledge levels surrounding the HPV, cervical cancer, and HPV vaccines. The educational component of the *HPV-Plus* program was key in increasing acceptability. Parents reported the lowest acceptability towards the program. This was found to be primarily due to perceptions of not being sufficiently engaged throughout program implementation. Increasing acceptability of HPV vaccination programs among key stakeholders is critical to facilitating vaccine uptake and meeting vaccination coverage targets. Our results demonstrate that the inclusion of a comprehensive education component within the *HPV-Plus* program was key in facilitating HPV vaccine acceptability amongst stakeholders.

## Background

Cervical cancer is the fourth most prevalent cancer among women globally and is a leading cause of cancer-related mortality worldwide [1]. Human Papillomavirus (HPV), which is a sexually transmitted virus, is a primary cause of cervical cancer and is responsible for over 90% of cervical cancer cases worldwide [1,2]. The global burden of cervical cancer is unevenly distributed, with sub-Saharan African countries having some of the highest cervical cancer incidence and mortality rates compared to any other region in the world [3–7]. Currently, East Africa has the highest cervical cancer incidence and mortality rates compared to any other region in the world, with over 40 cases per 100,000 of the population [3–5,8]. Moreover, sub-Saharan African countries also account for the highest HIV incidence rates globally [9]. The dual epidemics of HIV and cervical cancer is critical to note, as HIV significantly increases the risk of HPV infection and progression to cervical cancer [9,10]. The high and persistent burden of HIV in this region further heightens the unprecedented burden of cervical cancer, calling for immediate and tailored interventions addressing this growing burden [9].

Among countries in East Africa, Tanzania exhibits one of the highest cervical cancer morbidity and mortality rates, with cervical cancer ranking as the most frequently diagnosed cancer among women in Tanzania [11–14]. Current estimates indicate that over 10,000 women are diagnosed with cervical cancer annually in Tanzania, with over 6,525 deaths occurring each year [13]. HPV vaccines, which are approved for adolescents and young adults (aged 9–26 years), are highly effective in protecting against the high-risk HPV strains that cause cervical cancer (e.g., HPV 16,18, 31, 33) [15–17]. The World Health Organization (WHO) recommends the primary targets for HPV vaccination to be young adolescent girls aged 9–14 years before sexual debut [18]. Tanzania first introduced the HPV vaccine in 2014 through a two-year demonstration program supported by Gavi, the Vaccine Alliance [19]. This program demonstrated effectiveness and was successful in reaching high levels of vaccination coverage among adolescent girls. Given this program’s success, as of 2018, the Tanzanian Ministry of Health (MoH) introduced HPV vaccination for 14 year old girls into its national immunization schedule using routine strategies that emphasized school-based delivery supported by health facility and community outreach services [19,20].

Currently, the government of Tanzania has established targets to fully immunize 2.7 million adolescent girls and achieve vaccine coverage rates of 85% by 2025 [19]. To help achieve these goals, as well as to meet a broader range of public health needs for school-aged boys and girls, the Tanzanian MoH in collaboration with the Office of the President’s Office of the Regional Administration and Local Government (PO-RALG) developed *HPV-Plus*, an integrated service delivery program that packages HPV vaccines with complementary adolescent health services. This program was implemented with technical support from Jhpiego, a Johns Hopkins University affiliate organization, with Gavi funding. The *HPV-Plus* program was informed by human-centered design workshops in 2018, finalized in 2019, and contains a core package of services including age-appropriate HPV education, provision of deworming tablets, nutritional assessments, and visual acuity assessments. The implementation guidance allowed for integrated services to be delivered in three settings: health facilities, schools, and community outreach; however, it was particularly tailored to school-based vaccination days.

Nevertheless, the prioritized population of school-age girls, coupled with the two-dose vaccination schedule delivered six-months apart, poses unique challenges in successful HPV vaccination uptake [21]. Limited HPV vaccine knowledge and awareness, sociocultural issues (such as stigma associated with HPV being a sexually transmitted infection), and high rates of misinformation and misconceptions surrounding the HPV vaccine have contributed to low vaccine confidence, concerns about vaccine safety, vaccine mistrust, and increased vaccine hesitancy [4,22–25]. These factors may result in low acceptability of HPV vaccination programs, which are critical barriers to meeting established HPV immunization targets. Understanding vaccine acceptability and drivers of HPV vaccine hesitancy in target communities will be pivotal in improving vaccination uptake and coverage. Furthermore, the delivery of HPV vaccines using specific platforms that are targeted towards adolescents such as school-health programs, will be essential in meeting national targets.

The MoH, PO-RALG, and Muhimbili University of Health and Allied Science, in collaboration with Jhpiego and the Johns Hopkins Bloomberg School of Public Health International Vaccine Access Center, conducted a qualitative descriptive study to understand the acceptability of the *HPV-Plus* program, which was first introduced as a focused demonstration in two districts of Tanzania: Makambako (in Njombe Region) and Ubungo (in Dar es Salaam Region). These districts were selected in collaboration with MoH, PO-RALG and Jhpiego. The focused district demonstrations took place from November 2019 to March 2020; however, facilities were able to continue implementation after this period. For Makambako District, this was enabled by an extension of Gavi funding to allow implementation of *HPV-Plus* across Njombe Region. For Ubungo District, facilities were free to continue *HPV-Plus* with their own resources, as desired. The *HPV-Plus* program integrated the delivery of HPV vaccines with other selected adolescent health services. It comprised comprehensive health education for boys and girls aged 10–14 years, and provision of antiparasitic medicines (deworming), nutrition assessments, vision screening, and HPV vaccination for girls aged 10–14 years. We present findings from our qualitative study exploring the acceptability of the *HPV-Plus* program among various key stakeholders: program implementers, program planners, educators, adolescent girls, and parents. In assessing program acceptability, we aimed to understand stakeholder perspectives and attitudes toward the HPV vaccine, including knowledge and misinformation regarding HPV, cervical cancer, and HPV vaccines; as well as acceptability toward the integrated approach of *HPV-Plus*, and toward school-based service delivery compared to a health facility-based service provision. While a plethora of studies have been conducted assessing HPV vaccine acceptability in higher-income countries, less attention has been devoted to understanding HPV vaccine acceptability in low-income countries [26]. Additionally, studies that have sought to understand HPV vaccine acceptability in low- and middle-income countries do not typically include perspectives from a diverse array of relevant stakeholders, from program participants to planning/implementing stakeholders.

## Methods

### Theoretical framework

**Fig 1:**
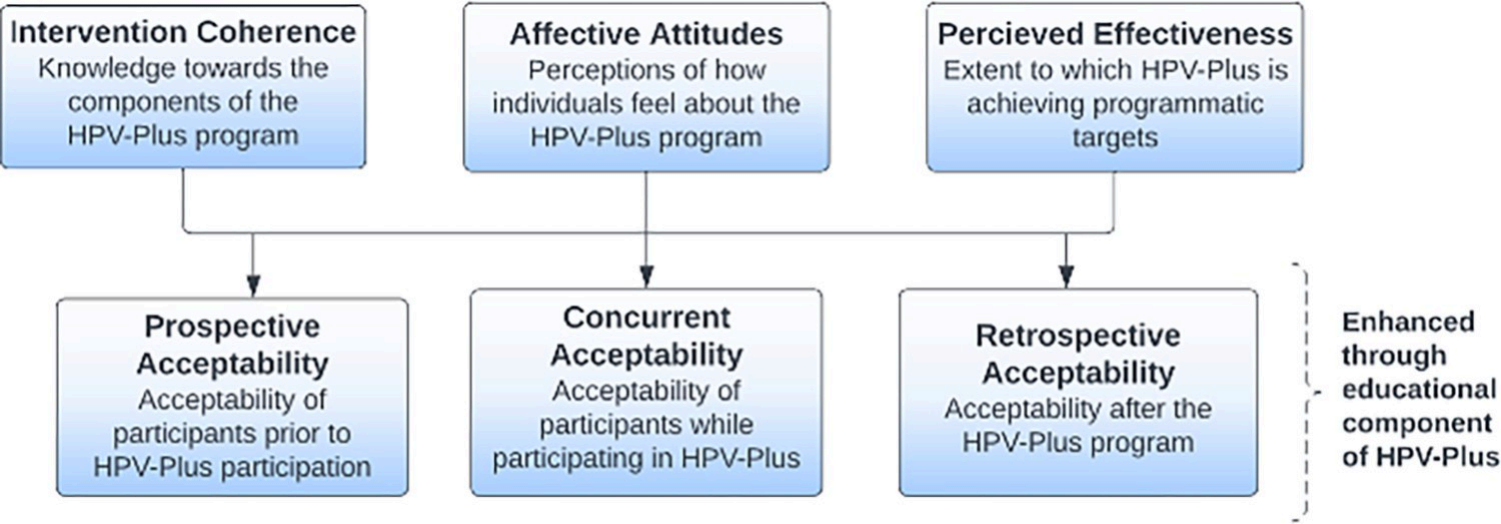
Theoretical framework of acceptability adapted for HPV-Plus.

The Theoretical Framework of Acceptability (TFA) guided our qualitative inquiry into the *HPV-Plus* program’s acceptability (Fig 1) [27]. TFA is used to guide the assessment of acceptability from the perspective of intervention implementers and participants. The framework posits that acceptability is not solely an attribute of an intervention, but a subjective evaluation made by individuals who experience or deliver an intervention, with distinctions between prospective, concurrent, and retrospective acceptability. TFA has been used in studies assessing the acceptability of healthcare interventions within development, piloting and feasibility, outcome and process evaluation [27,28]. In our study, we used the TFA constructs of affective attitudes (e.g. how participants feel about the intervention), intervention coherence (e.g. extent to which participants understand the intervention and how the intervention works), and perceived effectiveness (e.g., whether participants believe the intervention was effective) to evaluate acceptability of the *HPV-Plus* program among implementers and participants [27].

### Study setting, participants and timing

The Makambako District Council (Njombe Region) and Ubungo District Council (Dar es Salaam Region) had been selected for the focused demonstration of *HPV-Plus* as both districts needed to increase rates of HPV vaccine uptake, whilst representing a mix of urban (Ubungo) and rural (Makambako) settings. Study participants included program planners (e.g. Ministry of Health officials, Ministry of Education officials, Jhpiego, and PO-RALG Regional and District officials, procurement and logistics officials), program implementers (school health coordinators, educators, school officials, and health care providers), adolescent girls, and the parents of adolescent girls. While the age range for *HPV-Plus* was 10–14 years, interviews for this study took place over one year after *HPV-Plus* was implemented. Thus, a large percentage of adolescent girls were above the age of 14 at the time of the interviews. We included stakeholders of varying backgrounds to elicit a comprehensive overview of acceptability towards the HPV vaccine and the integrated *HPV-Plus* program.

### Recruitment

A list of eligible schoolgirls that received HPV vaccines through *HPV-Plus* was generated using registers that captured HPV vaccination coverage across implementation sites. With support from schoolteachers and trained providers, study staff contacted parents/guardians of eligible adolescent girls by telephone to provide information about the study and solicit permission to meet with the adolescent for an interview. Parents (i.e. mothers and fathers) and legal guardians who were approached regarding enrollment of their daughters’ in the study were also read informed consent documents to participate in a separate interview. Adolescent girls and her parent(s)/guardian(s) who agreed to participate in interviews were interviewed separately. Adolescents and their parent(s)/guardian(s) were still eligible to participate in the study even if one party in the dyad did not wish to be interviewed. Although adolescent boys participated in certain aspects of the HPV-Plus program, adolescent girls who were eligble to receive HPV vaccines were the priority in our study as this group received the broadest range of integrated health interventions including HPV vaccines.

Planning stakeholders and program implementers were recruited purposively through professional connections with Jhpiego Tanzania staff. Using stakeholder mapping activities with study staff, Jhpiego enumerated key personnel at various levels (national, regional, local) engaged with the planning and scale-up of the *HPV-Plus* program, immunization services, and other integration efforts supported by the MoH. Implementation and planning personnel were required to be living in or associated with the study communities and provided informed consent. Implementation and planning staff were categorized into one of the following categories: a service provider who provided integrated HPV services; a national or regional level program manager for Expanded Program for Immunization (EPI) and integrated services; a community or school official that was directly involved in the administration or implementation of the program; a person responsible for planning, policy design, and supervision of the project; or a person that was responsible for specific areas of interest related to the program (such as immunization, procurement, logistics, education, training).

### Data collection

Interview guides were developed through an extensive literature search of studies evaluating HPV vaccine program feasibility, acceptability, and sustainability in LMICs primarily within sub-Saharan Africa [23,29–31]. In-depth interviews (IDIs) with adolescent girls and parents explored perspectives on *HPV-Plus* program and included questions to evaluate program acceptability. These questions were accompanied by questions related to program feasibility and sustainability. The study received ethical review and approval from the Johns Hopkins Bloomberg School of Public Health International Review Board (Baltimore, Maryland, USA) and the National Institute for Medical Research (Dar es Salaam, Tanzania).

Interviews were conducted from June to July 2021, more than two years after *HPV-Plus* was introduced in Makambako and Ubungo Districts. Data collection was delayed due to local COVID-19 restrictions. Experienced data collectors fluent in English and Kiswahili were trained by an experienced in-country study coordinator with extensive experience in qualitative methods and interviewing techniques. Participants were able to select the interview location of their preference, and were interviewed by a trained data collector who was familiar with the communities from which participants were recruited. IDIs were conducted with program participants, which included adolescent girls and their parents, and other stakeholders such as implementation personnel and planning personnel. IDIs were conducted in English and/or Kiswahili, depending on participant preferences, and lasted 60 minutes on average. While the interviews were designed to specifically assess experiences from the focused demonstration period (November 2019 to March 2020), we accepted the reality that some participants would also report on extended implementation experiences.

### Data analysis

Interviews were audio-recorded and transcribed verbatim, with translations edited for clarity. Interviews that were conducted in Kiswahili were translated into English by a verified translator. Transcripts were analyzed using a team-based thematic analysis approach consisting of inductive (drawn from a review of transcripts) and deductive coding (extracted from interview guide questions). A set number of transcripts were selected for thematic analysis by DG, JGR, AG, and RL, in which an initial set of codes was developed deductively from the semi-structured interview guides. Following close reading of transcripts by investigators, codes were adapted or added to inductively incorporate emerging themes not explicitly addressed in the IDI field guides. After pilot testing and revising the codebook on a set number of transcripts, the codebook was programmed in Atlas.ti 8 (Scientific Software Development GmbH, Berlin, Germany), facilitating the application of codes to text segments across transcripts. Codes were then categorized into broader themes guided by TFA constructs. After coding was complete, nine random transcripts were chosen for intercoder consistency. A member of the study team that had not coded any of the nine transcripts served as the second coder and coded the nine selected transcripts. Intercoder consistency was estimated at 84%. After coding all transcripts, Baltimore-based study staff (JGR, DG, AG, RL, CM, KB, ZP) convened to identify the most salient insights emerging.

## Results

### Participant characteristics

A total of 87 participants were enrolled in the study (n = 25 adolescent girls, n = 16 parents of adolescent girls, n = 22 planning stakeholders, and n = 24 implementing stakeholders). Over half (n = 50) of participants were located in Njombe, and 37 were located in Dar es Salaam (Table 1). In evaluating the receptivity toward the *HPV-Plus* program, several themes emerged from the interviews. These themes included knowledge of the HPV Vaccine, perceptions of the *HPV-Plus* program, attitudes towards the *HPV-Plus* program, and the overall acceptability of the *HPV-Plus* program. Table 2 provides illustrative quotes in addition to the quotes highlighted within the narrative following the table.

**Table 1:** Stakeholders interviewed by group (n = 87).

Stakeholder Group	Region		
	Makambako District (Njombe Region)	Ubungo District (Dar es Salaam Region)	Total
Adolescent Girls	17	8	25
Parents/Guardians	11	5	16
Planning Stakeholders	12	10	22
Implementing stakeholders	10	14	24

**Table 2:** Additional stakeholder quotations.

DOMAIN	SOURCE	QUOTATION
**INTERVENTION COHERENCE: KNOWLEDGE AND MYTHS**		
	*Student in Njombe*	*“Some [students] said they were forbidden by their parents… They rejected some saying if they get vaccinated*, *they will not give birth*, *they will not be able to have children*.*”*
	*Parent in Dar es Salaam*	“*In the community people talk… they say the vaccine is not good… later it can affect girls after they got marriage it might affect their reproduction rates…they may become infertile*”
	*Registered Nurse in Njombe*	*“I think parents had wrong information that children might become infertile after vaccine*. *This was the most things that were mentioned there were no more negative effects mentioned than that*.*”*
**AFFECTIVE ATTITUDES AND PERCEPTIONS**		
	*Student in Dar es Salaam*	*"They said this here is important to vaccinate because it prevents this cervical cancer*, *so it is important to vaccinate so that it does not cause you problems later on when you become an adult*.* *.* *.*the benefit is to help you later to avoid complications…later we may not get cervical cancer*.*”*
	*Parent of student in Njombe*	*“I was shocked*, *the cervix*, *and she told me yes*, *I was surprised … It [my first impression] wasn’t good because if you look at the age of the child*.* *.* *.*”*
	*Health Teacher in Njombe*	*“I didn’t receive it really well…but after passing through various leaflets and go online to read we knew it is a good thing… this child should get it early so that it can be controlled early*.*”*
**PERCEIVED EFFECTIVENESS AND ACCEPTABILITY**		
	*Student in Dar es Salaam*	*It is [HPV vaccination] for my own good so I loved it*.* *.* *.*I liked it but I was scared*.* *.* *.*Injecting is not an easy thing*. *[I agreed to HPV vaccination] because they said it helps us women and I am one of the women here*.*”*
	*Parent in Njombe*	*" At first when I received the information*, *I thought maybe they are harmful because I have never been educated about this vaccine …When she [daughter]explained to me then I understood*.*”*
	*Education officer* in Dar es Salaam	*“I received it well*, *because it helped even our children to be aware of many things only*, *the education they were given was helpful because there are many issues that children do not know*.* *.* *.*now they included that they get even that education I received well*.*”*

### Overall acceptability

Program planners and implementers in their overall assessments found the *HPV-Plus* to be acceptable as a means to both prevent cervical cancer and address a broader range of adolescent health needs simultaneously. This overall acceptability was largely echoed by adolescent girls and parents. Within this high-level finding, many positive and negative details of nuance were brought out using our analytic framework, as discussed below. Additional quotes are provided in Table 2.

*Intervention coherence: Knowledge towards the HPV vaccine*

Across all stakeholder groups, education sessions were essential in dispelling misconceptions about the vaccine, increasing HPV vaccine and cervical cancer knowledge, and providing clarity to questions and concerns that participants had. Low HPV vaccine knowledge, coupled with high levels of misinformation and myths surrounding the vaccine, were cited across stakeholder groups as both preceding the program and persisting during program implementation. Myths included the HPV vaccine causing infertility, the vaccine resulting in illness and death, and increased sexual activity and promiscuity among vaccinated adolescents. Numerous participants across stakeholder groups reported having questions about the HPV vaccine before program implementation and voiced that the education sessions were effective in clarifying questions and concerns.

“*Others [in the community] say it can affect girls after they get married*…*it might affect their reproduction rates*…*I was angry [when hearing about the program] because of these street reports…they are injecting needles in those schools so that children do not give birth later"-* Parent, Njombe

Nearly all adolescent girls who were interviewed stated that they had little to no information on HPV vaccines prior to the program. Education on cervical cancer, HPV vaccines, and dosing schedules were reported to improve students’ knowledge on the disease process of cervical cancer and the benefits of vaccination for prevention. In addition, students were provided with comprehensive sexual and reproductive health education. Group education in the school-based program was especially well-received by students and often distinguished as the element they liked most in the integrated program. This was confirmed by program implementers with several stating that providing educational sessions in a group setting encouraged students to ask questions regarding the vaccine, with numerous students requesting more information after educational sessions. Several students reported hearing about myths regarding the HPV vaccine within the larger community, with many of these students believing these myths prior to receiving education.

“*I remember before the vaccination we didn’t understand anything*, *but after the vaccination*, *that’s when they told us this vaccine is going to be again on the tenth month*, *and make sure you have to finish the dose because if you inject one you have not finished*.*” -* 15-year-old student, Njombe

For many parents, HPV vaccine knowledge prior to the *HPV-Plus* program stemmed from what they heard about the HPV vaccine from others in their local communities, which consisted largely of rumors and misconceptions. Parents cited low knowledge levels towards the HPV vaccine, with several parents stating that they believed certain rumors about the HPV vaccine prior to receiving accurate information. The most cited misconception among parents was that the HPV vaccine caused infertility. Several parents highlighted the need for more education within the community to dispel myths and misinformation.

“*What shocked me was just that street education because everyone was talking about it*, *I was just scared that they said those who were vaccinated would not be able to give birth again*, *that is when I was hurt*.*”–*Parent, Njombe

Program implementers received information on the *HPV-Plus* program and the benefits of HPV vaccination prior to program implementation. Information and education on the *HPV-Plus* program was provided to program implementers weeks to months in advance before actual program implementation. Several program implementers had low prior knowledge of HPV vaccines, and voiced questions about the purpose of HPV vaccines prior to receiving such education. A number of program officers also believed myths about the HPV vaccine prior to receiving education through *HPV-Plus*.

“*I had a lot of questions…they want to reduce the population of Africa… these children are likely to mature so that at the end of the day they have less fertility and do not reproduce much*, *but after getting that education I understood that even I as an adult am supposed to check whether I have it [cervical cancer]*.*”–*Health teacher, Njombe

### Affective attitudes and perceptions towards the HPV-Plus program

The majority of adolescent girls described positive feelings towards HPV vaccines and the *HPV-Plus* program, particularly after receiving health education. Education was found to be beneficial in not only increasing knowledge, but also fostering positive attitudes towards the HPV vaccine among students. Several adolescent girls stated they were fearful of the vaccine due to the pain associated with injections. However, despite this fear, adolescent girls voiced the importance of undergoing vaccination to reduce their future risk of cervical cancer. Students noted that being surrounded by peers was helpful in overcoming initial fears of inoculation.

“*It is [HPV vaccination] for my own good so I loved it*… .*I liked it but I was scared*…*injecting is not an easy thing*. *I agreed to HPV vaccination because they said it helps us women and I am one of the women here*.*” -* 15-year-old student, Dar es Salaam

Parents’ perceptions and attitudes towards the HPV vaccination program varied substantially. Positive attitudes towards the program were reported by parents as due to the receipt of trustworthy information on the HPV vaccine and the overall program. Several parents stated that they viewed the vaccine as important in improving health outcomes for their children; and were more likely to endorse positive feelings towards the program if they had known that stakeholders such as the government officials, teachers, and health care providers were involved. However, many parents stated they were skeptical of the program and held negative beliefs due to not having adequate information, and not being provided with education about the benefits of HPV vaccination prior to program implementation. There were inconsistencies in the education that was received among parents, with some parents being contacted by program officials to attend information seminars, while other parents stated they were not informed about the program. Parents who felt they did not receive adequate information did not perceive the vaccine to be beneficial and demonstrated hesitancy towards their children being vaccinated.

“*I received it [HPV vaccination] well by one hundred percent because we did not see any child who had problems with this vaccine*, *they are all safe*. *After the seminar I educated her to go and get this vaccine*…*I want my baby to be healthy and not have effects that will cause problems later*.*”–*Parent, Dar es Salaam

“*I think teachers were the one who called us parents*, *the children were given leaflets that we need you parents at the school…when we arrived they started giving us the seminar…now when we go to the school we found teachers and specialists they are saying this vaccine is to prevent cervical cancer…and we as parents always listen to specialists mostly”–*Parent, Njombe

Similar to the other key informant groups, program planners and implementers were more likely to demonstrate positive attitudes towards the *HPV-Plus* program after being provided with education on the purpose and benefits of the HPV vaccine. Program planners and implementers cited having positive feelings and attitudes towards the program in knowing that HPV vaccination would be beneficial in reducing cervical cancer outcomes and disease burdens in the community. Among planners and implementers, teachers more often reported negative perceptions and attitudes towards HPV vaccines and the *HPV-Plus* program prior to receiving education.

“*I was shocked…It’s not like I didn’t receive it [the HPV-Plus program] well*, *[however] I always have to ask*, *that is…about its effects*, *its benefits…these are someone’s children and we live with them for four years here*. *Maybe she is married only two or three years she has not given birth [because] you vaccinated her*. *So I wanted to satisfy myself [through looking up information]*, *after satisfying myself I said it was okay*.*”—*Health teacher, Njombe

### Perceived effectiveness and acceptability of the HPV-Plus program

Across stakeholder groups, parents exhibited the most hesitancy towards the *HPV-Plus* program, which was primarily attributed to the lack of accurate knowledge about the program. Based on their beliefs, it was not uncommon for some parents to forbid their children from getting vaccinated. Lack of awareness and engagement throughout the program implementation process was a prominent complaint among parents.

“*There was no information [provided on cervical cancer]*. *It was just a conversation between me and my daughter*, *we were not called as parents to say we had a seminar*.*”–*Parent, Njombe

Parents who did exhibit high program acceptability stated that the health benefits associated with HPV vaccination were important to them. Although the program included additional adolescent health services, most parents seemed to either lack accurate information on the complementary services or were unaware that additional health services were provided alongside HPV vaccine. Parents who were aware of the supplementary adolescent health services voiced being satisfied with the services provided.

“*I was happy because my child told me that they were given medication and her eyes were checked*, *and she later received the vaccination*.*”–*Parent, Njombe

Students reported feeling supported and empowered to participate in the program. As with parents, a key rationale for program acceptability was the benefits of the HPV vaccine in preventing cervical cancer. Several adolescent girls noted that some of their peers did not participate in the program due to parental refusal. Adolescent girls who demonstrated high acceptability towards the program also voiced encouraging and supporting their peers to participate in the program.

“*I persuaded my friends to go and inject*, *I was the one who persuaded them after I was inspired*.*”–* 15-year-old student, Dar es Salaam

Program planners and implementers had high acceptability overall of the program and voiced that the *HPV-Plus* program effectively met HPV vaccination targets, particularly as HPV vaccination rates were low prior to program implementation. Program planners and implementers highlighted the efficacy of the *HPV-Plus* program filling in gaps between education and the uptake of health behaviors, with stakeholders at multiple levels effectively coordinating program implementation to ensure that coverage targets were met. It was noted that collaboration with education officers was critical in heightening acceptance and subsequent uptake of HPV vaccines. Regarding recommendations for increasing acceptability, nearly all program implementers voiced that there was a need for accurate information regarding the vaccine as levels of misinformation tended to be high among parents.

“*To my personal understanding of this service to me I found it to be good*, *because this integrated service was helping a ten- to fourteen-year-old girls*. *The biggest challenges we faced in high schools*, *especially these private schools…every time you go for a service*, *we did not get much cooperation from the principal and teachers*. *They used to think that if you have these vaccines they will be unable to give birth*. *So we did not get enough cooperation*. *I think later after providing adequate education and asking for the numbers of the parents of the children*, *because it is a boarding school*, *the problem was solved and we were able to cooperate well*.*”–*Special education officer, Njombe

“*It [coverage] increased because we involved the ward education officer of the primary schools*, *CBGC [Community-based Group Coordinators]*, *peer educators*, *district medical officers*, *religious leaders*, *mayors*, *teachers and the Regional Commissioner*. *We talked about it with all our might and it was a topic everywhere and that’s why we ascended the charts*.*”*–Program officer, Dar es Salaam

### Acceptability of school-based delivery compared to use of health-facilities

Across districts, schools were the preferred venue for HPV vaccine services, as they were viewed as being ideal settings for targeting adolescents in large numbers. Students reported feeling supported and empowered by teachers to participate in the program. Group education in the school-based program was especially well-received by students and often distinguished as what they liked most about the program. Students enjoyed receiving the education alongside their peers and felt comforted and supported by one another, particularly in overcoming initial fears of inoculation.

“*In the whole group*, *you may be educated*, *even if someone asks a question you can consult with your colleague here how to ask*, *as opposed to an individual*.*”–* 15-year-old student, Njombe

Parents stated that school-based delivery offered convenience and reduced several logistical challenges (e.g. transportation accessibility and costs, loss of wages due to having to take time off work, costs of health care visit, childcare challenges, long wait times at health facilities). Parents who were employed emphasized difficulties taking time off work to take their children to health facilities to receive HPV vaccination.

“*Near my home I have a business*, *so I can just let someone watch for me and go to the school that is close by*… *but if you are told to come to Kimara (the hospital) you see the challenge… I have to close the shop until evening*.*”–*Parent, Dar es Salaam

Program implementers and planners expressed high acceptability of school-based delivery and disclosed that school-based delivery allowed for more effective outreach, resulting in more success in meeting vaccination targets. Program implementers and planners also cited benefits in school-based delivery reducing costs and being more inclusive through engaging multiple stakeholders throughout the community. Commenting on the benefits of school-based delivery, stakeholders said that health facilities do not offer the physical space required to speak with adolescents privately about sexual and reproductive health topics including HPV vaccines.

“*Ubungo is very large*… *We were very low in vaccination coverage*, *our coverage was very low but we questioned how can we be able to involve those young people but not only to involve young people but also to share with other departments for example education*. *At first there was a gap between education and health…but the campaign integrated it*. *We came*, *sat together and teachers we planned together and performed*. *We performed well so that also help to reach the targeted us in a way that is simple but that it is participatory*.*”-* Ministry of Health official, Dar es Salaam

“*At health facilities…we have no place to talk to these young people because even if we go to school we tell them if there is anything you want to know more should come here at Sinza hospital but as you can see for yourself here on the benches it was full in there it was full so this youth cannot open up because there is no friendly environment for them to open up…first the area should be set aside for youth service for example we provide youth services in a certain room and when they come here to our center we have a special area for them”*–Health care worker, Dar es Salaam

A subset of parents and girls identified health facilities as more fitting sites for health care, and the potential for a broader range of non-vaccine services to be integrated. A number of respondents identified the need for health facilities to be more responsive to adolescent preferences and needs if they are to become sites for *HPV-Plus* to be implemented on a greater scale.

“*…at the hospital most people go to get different services so it will be easier for most people to get that service [HPV vaccines]…”*–Parent, Njombe

“*I know at the health facility*, *I would have trust more than at schools where they just explain then you go for test…”*—Parent, Njombe

## Discussion

Improving HPV vaccine acceptability can heighten demand generation which can be critical in successfully meeting national immunization targets [32]. Our qualitative study explored knowledge, attitudes, perceptions, and acceptability toward an integrated school-based HPV vaccination program for adolescent girls across two districts in Tanzania. The acceptability of the *HPV-Plus* program was largely rooted in knowledge levels regarding the vaccine and the disease it was preventing. The majority of participants in our study reported that prior to the *HPV-Plus* program they had low levels of knowledge toward HPV vaccines, HPV, and cervical cancer. This is critical to highlight as knowledge gaps have been shown to contribute to major barriers to the uptake of HPV vaccines in numerous settings [22,33]. Education was crucial to fostering positive affect and attitudes toward HPV vaccines in our study, a finding which has been mirrored in other countries throughout sub-Saharan Africa [23,34–36]. The importance of education in improving HPV vaccine knowledge and acceptance, while reducing vaccine hesitancy, has been demonstrated in vaccine introduction programs throughout other regions of Tanzania alongside other countries within sub-Saharan Africa [34,37–40]. Participants in our study reported positive attitudes regarding the *HPV-Plus* program particularly after receiving health education on the HPV vaccine. Positive attitudes were found to be a facilitator in HPV vaccine acceptability and uptake among adolescents. Similar findings have been reported in studies assessing HPV vaccine acceptability among beneficiaries [25,41].

Beyond knowledge, education must also address misinformation and rumors regarding the HPV vaccine. These were prevalent in the communities where the *HPV-Plus* program was implemented, and were voiced not only among adolescents and parents, but also voiced among program implementers. This highlights the need for educational initiatives to be targeted towards multiple stakeholders: parents, adolescents, health workers, teachers, and program officials, to support the introduction of HPV vaccination programs particularly within school-based settings [42]. Addressing HPV vaccine myths and misinformation was found imperative to increase vaccine uptake and program acceptability in our study, especially as the sexual and reproductive health context of HPV and cervical cancer can result in heightened stigma, which can perpetuate misinformation [22,25,43]. Misinformation is a well-documented contributor to negative vaccine attitudes, thus necessitating the development of interventions that build high levels of knowledge and awareness; whilst supporting positive attitudes as this is important for addressing vaccine acceptability [22]. Across stakeholder groups, parents were of particular concern. They reported the lowest acceptability towards the *HPV-Plus* program as well as persisting knowledge gaps. There appeared a clear need for more educational messages in addition to other opportunities to be more directly engaged within the program. Studies that have assessed HPV vaccination program acceptability in both high-income countries and low- and middle-income countries (LMICs) have noted that positive attitudes of parents towards HPV vaccination is a strong predictor of HPV vaccination uptake by their children [30,40,44–46]. Interventions must place an emphasis on educating parents and caregivers in order to increase HPV vaccine acceptability and uptake among adolescents [22,44]. Key messages that promote HPV vaccines through culturally tailored, gained-framed appeals must be communicated consistently and mutually reinforced in order to heighten program acceptability and facilitate the uptake of HPV vaccines [47,48].

The integration of other adolescent health services with HPV vaccination in school-based settings was positively received across stakeholder groups. A number of recent studies have reported that school-based delivery in LMICs is often preferred and has demonstrated high acceptability and efficacy, particularly when there is adequate engagement of key stakeholders during the pre-implementation phase[21,49,50]. Most participants in our study voiced preferences for school-based delivery of HPV vaccinations compared to health facilities, as it significantly mitigated logistical burdens, reduced program-associated costs, while allowing adolescents to learn simultaneously with their peers. These findings are consistent with that of other studies assessing school-based delivery platforms for HPV vaccination where it has been reported that school-based delivery is effective in increasing vaccination acceptability and uptake within communities [51–55]. School-based platforms for HPV vaccine delivery provides an opportunity to link HPV vaccines within integrated packages for adolescents[4,29,56]. Participants endorsed the benefits of having integrated services within the *HPV-Plus* program. Previous studies evaluating delivery strategies for HPV vaccines in LMICs have noted that adolescents have a wide array of health needs that can potentially be met through offering full-range services such as deworming, vision and hearing screening, sexual and reproductive health education, access to feminine hygiene products, mental healthcare, and vaccination services[32,55]. The provision of such integrated packages within schools may be more appealing to adolescents given the inclusion of complementary health services within a setting of substantial familiarity[29,38,57]. A subset of parents and girls identified ways that health facilities could be made more responsive to adolescent needs and preferences. Increasing the volume of the “youth voice” in the design of services, both for vaccination and linked services, has been identified as an area of future attention by global commentators and researchers working on *HPV-Plus* [55]. Various HPV immunization demonstration projects throughout sub-Saharan Africa have indicated higher levels of final dose coverage rates when schools are used as a delivery venue compared to standard health care facilities [4,47,58]. Thus, the integration of adolescent health services with HPV vaccination and developing school-based delivery platforms may increase efficiency in meeting national targets.

### Limitations and future recommendations

Our study is not without limitations. Given our study methods, the findings may not be generalizable to other settings; however, our results provide insight on the factors that contribute to HPV vaccine acceptability within low-resource settings in Tanzania. Although the *HPV-Plus* program included a broader age range of adolescent boys and girls in comprehensive education, this group was not interviewed in our study due to resource limitations. Additionally, our study did not include perspectives of other key members who may have influenced the acceptability of the *HPV-Plus* program (e.g. community leaders, religious leaders). This omissions, and the recruitment of adolescent girls who had received vaccination, may have contributed to selection bias, and important perspectives regarding the program may not have been captured. Despite these limitations, the sample in our study consisted of a diverse array of stakeholders, which facilitated data source triangulation which allowed the researchers to gain diverse perspectives and reinforcement of the credibility of the qualitative responses. In addition, there is a possibility that social desirability was present when participants provided responses that would be deemed favorable by interviewees. The time between the focused demonstration and our interviews may have affected participants’ recall, and some participants may have related their answers to experience of the scaled-up implementation rather than the original pilot; we do not see this as likely to bias toward either positive or negative associations.

As HPV vaccination programs continue to expand throughout Tanzania and other countries in sub-Saharan Africa, future studies are needed to identify best practices in improving program acceptability among stakeholders at all levels. Recently, the WHO Strategic Advisory Group of Experts on Immunization has recommended one-dose of HPV vaccines in LMICs after evaluating evidence that a single-dose schedule provides comparable efficacy to that of a two-dose regimen [59]. Although this recommendation may reduce substantial barriers in meeting vaccination coverage targets, there remains an urgent need to better understand vaccine acceptability and hesitancy, coupled with strategies to increase acceptability as this may pose as a barrier to achieving coverage targets. Studies should consider the development of effective strategies for community mobilization along with communication campaigns that are tailored to the context in which the program is being implemented. Our study demonstrated high acceptability with school-based delivery of HPV vaccines; however, future studies should consider how to reach out-of-school girls, particularly in settings where school drop-out rates are high, and how to develop health facilities that are more responsive to adolescent needs and preferences. Future studies must also be conducted to explore acceptability of HPV vaccines for out-of-school girls as levels of acceptability may significantly differ between those who are in school compared to out of school. As more countries place increased focus on developing gender neutral programs that target both adolescent boys and girls for HPV vaccination, it will be critical to garner the perspectives of adolescent boys and explore their perspectives related to vaccine acceptability.

## Conclusion

Tanzania’s *HPV-Plus* program was found to be acceptable overall by all stakeholders, both in provision of HPV vaccination and in its approach to integration of other adolescent health services. The comprehensive education component was reported as the most important contributor to acceptability and in driving uptake. Of all stakeholders, it was the parent group that requires greater involvement in education and program operations. Increasing HPV vaccine acceptability across all relevant stakeholders, including parents, students, community leaders, program planners, and program implementers will be critical in driving the success of the *HPV-Plus* program nationwide within Tanzania. Comprehensive health education that involves stakeholders at all levels must be prioritized to successfully heighten acceptability and uptake of HPV vaccines among communities. The school-based delivery of *HPV-Plus* was an acceptable means to integrate complementary adolescent health services, was seen to be more effective in reaching a higher proportion of adolescents and may function as a trusted service delivery platform that fosters confidence among participants.

## Supporting information

S1 File HPV vaccine integration tanzania—interview guides. (DOCX)

S1 Questionnaire Inclusivity in global research questionnaire. (DOCX)

S1 Data Participant data. (XLSX)
