# Supplementary material for: Acceptability of an integrated school-based HPV vaccination program within two districts of Tanzania: A qualitative descriptive study
Source: PLOS Glob Public Health. 2023 Jan 4;3(1):e0001394. doi: 10.1371/journal.pgph.0001394 (PMC10021529; doi:10.1371/journal.pgph.0001394)
Supplement: S1 File — (DOCX) [file pgph.0001394.s001.docx]

**FGD/IDI Guide for Stakeholders (e.g.: Health Workers, Teachers)**

*Feasibility:*

1. Please describe in your own words what services were offered through this new program
2. What work was required of you, to prepare for the new program? To deliver the integrated services? How much of your time does providing the integrated services require of you? How does that compare to the way you used to deliver similar services?
3. Does it make sense to keep offering the integrated services? Why/why not?

*Acceptability****:***

1. What was your first opinion, your first thought, when you heard about plans for this new program?
   1. *Probe:* Were you concerned? Excited? Pleased? Worried?
   2. Has your opinion changed?
2. In your words, what are the most important needs of the adolescent girls who come to (this clinic/this school/live in this community)?
3. Did this integrated program (of HPV vaccine with other services) meet those needs?
   1. What parts of the integration did you think were successful?
   2. What parts were not?
4. How do you think this program has affected the community?
   1. *Probe:* the adolescent girls? Parents? Who else?
   2. Were there benefits? Unintended negative consequences?
5. How do you think this program has affected the (health facility/government/school?)
   1. Was there a big impact? A little impact?

*Sustainability****:***

1. Should this program be continued into the future?
   1. *Probe:* Should it be changed? Discontinued? Why/why not?
2. In the future, do you think it will it be possible to continue this program here?
   1. *Probe:* Why or why not? Funding? Resources? Training? Supportive supervision?
3. From your perspective, what would need to happen for the program to be continued successfully?
   1. *Probe:* More funding? Supplies? Personnel? Policies? Procedures?

*Cost:*

1. While you have been providing integrated services as part of the pilot we are evaluating, on average how many hours per day do you spend on
   1. HPV immunization related tasks?
   2. HIV related services?
   3. ASRH related services?
2. What is your monthly average of income from providing these integrated services during the pilot?
3. How many hours do you spend working or delivering health services as part of the pilot at
   1. School-based facility?
   2. Community-based facility?
   3. HIV clinics?
   4. ASRH clinics?
4. Compare to in the now, I’ll ask you to answer those same questions for the past prior to the integrated services
5. At which facility were you working or delivering health services?
6. How many hours per day on average do you spend on HPV immunization related task?
7. How many hours per day on average do you spend on HIV/ ASRH related services?
8. What was your monthly average of income prior to this pilot program?

**Additional Questions for HIV service providers:**

1. In your opinion, did the program do enough to protect the privacy and confidentiality of these girls?
   1. *Probe:* Was HIV status kept in proper confidence? How/how not?
2. In your opinion, did the program promote a safe environment for adolescent girls living with HIV to participate? How / How not?

**FGD/IDI Guide for Direct Beneficiaries (adolescent girls)**

*Feasibility Questions*

1. As we discussed already, you got the HPV vaccine with other services. Could you describe the other services, in your words?
2. In the past, why would you go to clinic or to see a medical provider?
   1. *Probe:* Reproductive health? Vaccines?
   2. Was it easy to get services you needed? Difficult?
   3. How long did you have to travel?
   4. How did you travel?
   5. Did you have to miss school / work / other activities?
3. Compared to in the past, I’ll ask you to answer those same questions for the more recent services you got *(Interviewer: read again through the same questions listed under Question 2)*
4. How did this time, getting the vaccine with other services, compare to the past, when you went to clinic or a medical provider?
   1. Anything better? Easier? Worse? Harder?
5. Did you take the vaccine? Which of the other services that were offered did you accept?
   1. What influenced you to take all the services, or to take only some of them?
      1. *Probe according to response:* What were the reasons? Would anything have made you more likely to take all of the services? Less likely?

*Acceptability & Sustainability Questions*

1. Why did you decide to participate in this program?
   1. Probe: Did others influence you? Parents? School teacher? Friends? Someone else?
   2. How did you first hear about the program?
2. What would make you want to come back for the second vaccine and services?
   1. *Probe:* Not having to miss school? Work? Family being more supportive? More time? Expense?
3. What did you like about the program?
   1. *Probe:* Did you have a favorite part? Why?
4. What didn’t you like about the program?
   1. *Probe:* Did you have a least favorite part? Why?
   2. How would you change that part? How would changing it improve your experience?
5. I’m interested in knowing about the feelings you had when you were participating in the program:
6. *Probe:* Did you feel safe? Comfortable? Did you feel like the program protected your private information?
   1. Why or why not?
7. Would you tell anyone else to come for these services? (*Probe:* Who else – friends, sisters, cousins? Would they want to come?)

**FGD/IDI Guide for Indirect Beneficiaries (Parents/Guardians)**

*Feasibility Questions*

1. As we discussed already, your daughter/ward got the HPV vaccine with other services. Could you describe the services she was offered, in your words?
   1. Did she accept all of them? *Probe:* Which ones? Why/why not?
2. In the past, why would your daughter/ward go to clinic or to see a medical provider?
   1. Was it easy to get those services? Difficult?
   2. How long did you and your daughter(/ward) have to travel to get the services?
   3. How did you travel? *Probe*: on foot, motorcycle, taxi, etc.
   4. How much did the transportation cost?
   5. Did your family pay any of your own money for the HPV vaccine and other services?
   6. Did you miss work / other activities? If so, how many hours?
   7. Did you lose out on any income in order to bring your daughter(/ward) for these services?
   8. What is your monthly average total income?
3. Compared to in the past, I’ll ask you to answer those same questions for the more recent services she got *(Interviewer: read again through the same questions listed under Question 2)*
4. How did this time, getting the vaccine with other services, compare to the past, when you went to clinic or a medical provider?
   1. Anything better? Easier? Worse? Harder?

*Acceptability & Sustainability Questions*

1. Why did you decide to have your daughter get these services?
   1. Probe: Did others influence you? Parents? School teacher? Friends? Someone else?
   2. How did you first hear about the program?
2. What would make you want to have your daughter come back for the second vaccine and services?
   1. *Probe:* Not having to miss school? Work? Family being more supportive? More time? Expense?
3. Would you recommend to anyone else to bring their daughters for these services?
